# Cerebrovascular disease and stroke

**DOI:** 10.1136/adc.2008.142836

**Published:** 2008-06-30

**Authors:** J Pappachan, F J Kirkham

**Affiliations:** 1 Departments of Paediatric Intensive Care and Paediatric Neurology, Child Health Directorate, Southampton General Hospital, Southampton, UK; 2 Institute of Child Health, London, UK

## Abstract

Stroke and cerebrovascular disorders are important causes of morbidity and mortality in children; they are already amongst the top 10 causes of childhood death and are probably increasing in prevalence. Acute treatment of stroke syndromes in adults is now evidence based. However, paediatric stroke syndromes are far less common and the differential diagnosis is very wide, but the individual health resource implications are much greater because of the life-long treatment costs in survivors. Recognition and consultation with a paediatric neurologist should be rapid so that children can benefit from regional services with emergency neurological, neuroradiological and neurosurgical intervention and paediatric intensive care. This review focuses on the epidemiology, presentation, differential diagnosis, generic/specific emergency management and prognosis of acute stroke in children. Its aim is to educate and guide management by general paediatricians and to emphasise the importance of local guidelines for the initial investigation and treatment and appropriate transfer of these children.

Stroke and cerebrovascular disorders are important causes of morbidity and mortality in children; they are already amongst the top 10 causes of childhood death and are probably increasing in prevalence. Recent epidemiological data suggest incidence rates of 2–5/100 000 children/year for childhood stroke (at least 300 a year in the United Kingdom),^1^ with a peak in the first year of life. Boys are at higher risk than girls. The incidence may have increased over the last 25 years as a consequence of increased recognition, less invasive vascular diagnosis (MR/CT angiography) and therapeutic advances allowing children with predisposing conditions (eg, congenital heart disease, anaemias, malignancy, meningitis) to survive. However, this trend may be offset by screening and primary prevention strategies in those at risk (eg, with sickle cell disease, (SCD).^2^ ^3^ Neonatal stroke is more common, with an incidence of up to 63 per 100 000 live births.^4^ Health care utilisation is not adequately reflected by incidence figures but is more appropriately described by disease outcome. As well as a 20% mortality and a high recurrence rate, at least half of the survivors of these events have permanent cognitive or motor disability.^1^ ^2^ ^4^ Rapid recognition^5^, investigation^6^ ^7^ and appropriate management^5^^–^8 (boxes 1 and 2, figs 1–5) should improve these disappointing figures.

**Figure 1:**
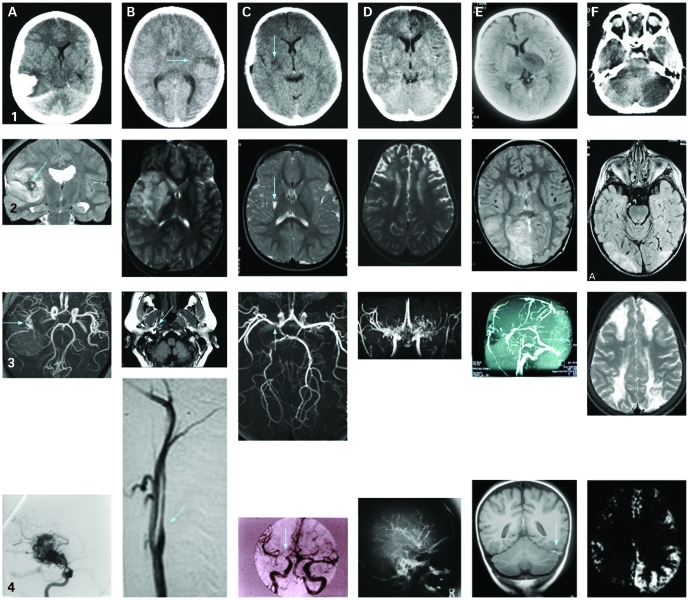
Neuroimaging in children with stroke. Row 1: CT scans; row 2: T2-weighted MRI scans; row 3: vascular imaging (A–D: magnetic resonance angiography (MRA); E: MR venography (MRV); F: watershed infarction); row 4: additional imaging which may be useful in difficult cases (A–D: conventional angiography; E: venous thrombosis on MRI; F: potentially reversible signal abnormality on diffusion weighted imaging). Column 1: haemorrhage; column 2: extracranial dissection; column 3: transient cerebral arteriopathy; column 4: moyamoya; column 5: venous sinus thrombosis; column 6: posterior circulation stroke and stroke mimics – posterior leukoencephalopathy, “covert” watershed ischaemia, hemiplegic migraine. A: Haemorrhage; A1: spontaneous intracerebral haemorrhage with midline shift; A2: MRI showing haemorrhage from mycotic aneurysm in a patient with subacute bacterial endocarditis; A3: mycotic aneurysm on MRA; A4: conventional arteriography showing arteriovenous malformation. B: Dissection; B1: infarct in a child who had suffered a minor head injury 24 h before; B2: large cerebral infarct after head injury; B3: fat-saturated T1 MRI of the neck showing haemorrhage in the vessel wall; B4: conventional arteriography showing tapering “rat’s tail” appearance characteristic of extracranial dissection. C: Transient cerebral arteriopathy; C1: infarct in a child with stuttering stroke onset; C2: infarct in a child with recent varicella; C3: short segment of middle cerebral artery stenosis (MRA from child in C2); C4: conventional arteriography from child in C1 showing longer segment of middle cerebral artery stenosis (the infarct had extended in size on the post-arteriography CT scan). D: Moyamoya; D1: bilateral frontal infarction in a child with livedo reticularis; D2: bilateral frontal infarction in a child with sickle cell anaemia; D3: bilateral middle cerebral artery stenosis with collateral formation obvious on MRA; D4: conventional arteriography showing attenuation of major intracranial vessels and collaterals. E: Venous sinus thrombosis; E1: bilateral thalamic signal change in severe iron deficiency anaemia; E2: occipital signal change in nephrotic syndrome; E3: sagittal sinus thrombosis in systemic lupus erythaematosus presenting with psychiatric symptoms; E4: transverse sinus thrombosis on plain MRI in child in E1. F: Posterior circulation stroke and stroke mimics; F1: cerebellar infarction in a boy with vertebral dissection; F2: bilateral occipital signal change suggestive of posterior leukoencephalopathy; F3: bilateral watershed infarction after facial infection in sickle cell anaemia (MRA and MRV were normal); F4: diffusion-weighted imaging shows potentially reversible pathology in a patient with hemiplegic migraine and normal T2-weighted MRI.

**Figure 2:**
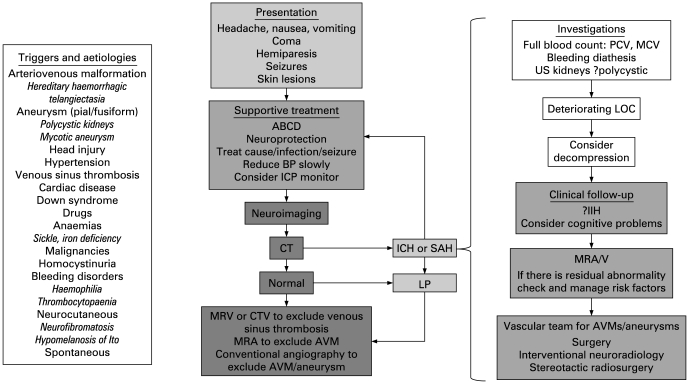
Flow diagram for the diagnosis and management of haemorrhagic stroke. ABCD, airway, breathing, circulation, disability; AVM, arteriovenous malformation; BP, blood pressure; CT, computed tomography; CTV, CT venography; ICH, intracerebral haemorrhage; ICP, intracranial pressure; IIH, idiopathic intracranial hypertension; LOC, level of consciousness; LP, lumbar puncture; MCV, mean cell volume; MRA, magnetic resonance angiography; MRV, magnetic resonance venography; PCV, packed cell volume; SAH, subarachnoid haemorrhage; US, ultrasound.

**Figure 3:**
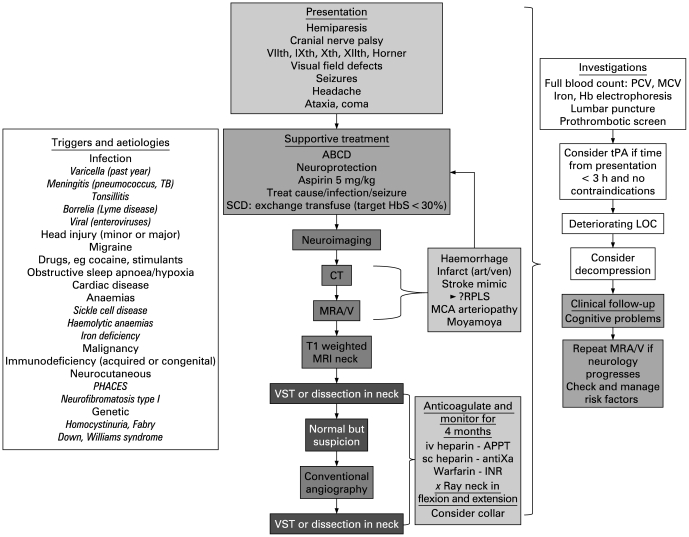
Flow diagram for the diagnosis and management of haemorrhagic stroke. ABCD, airway, breathing, circulation, disability; MCA, middle cerebral artery; MRA, magnetic resonance angiography; MRV, magnetic resonance venography; PHACES, posterior fossa malformations, haemangiomas, arterial anomalies, cardiac defects, eye abnormalities, and sternal or ventral defects; RPLS, reversible posterior leukoencephalopathy syndrome; SCD, sickle cell disease; VST, venous sinus thrombosis.

**Figure 4:**
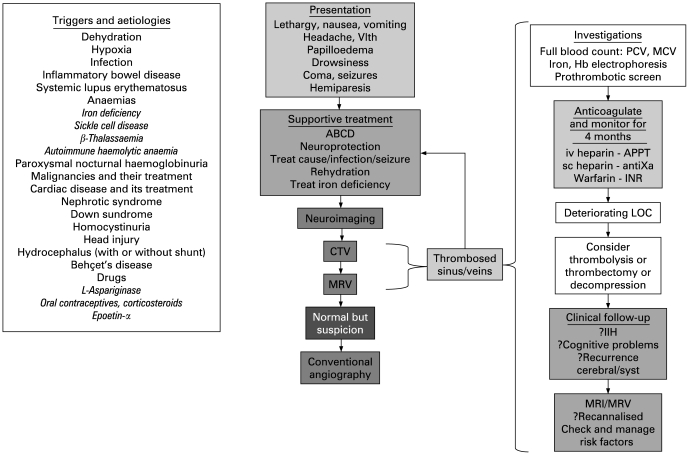
Flow diagram for the diagnosis and management of venous sinus thrombosis. IIH, idiopathic intracranial hypertension. ABCD, airway, breathing, circulation, disability; CTV, computed tomography venography; LOC, level of consciousness; MRV, magnetic resonance venography.

**Figure 5:**
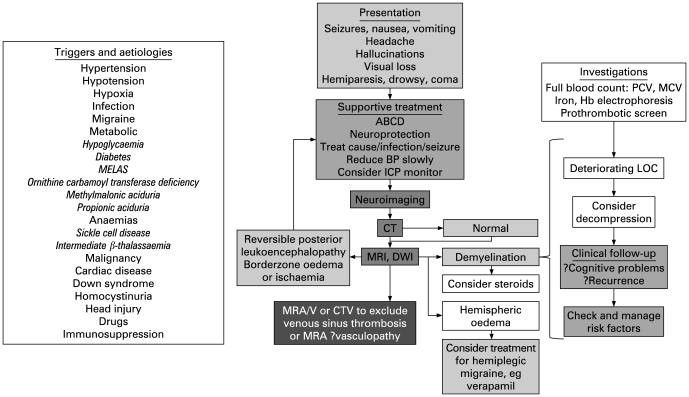
Flow diagram for the management of stroke mimics. ABCD, airway, breathing, circulation, disability; BP, blood pressure; CTV, CT venography; DWI, diffusion weighted imaging; LOC, level of consciousness; MRA, magnetic resonance angiography; MRV, magnetic resonance venography.

Haemorrhagic stroke (figs 1A, 2) includes intracerebral haemorrhage (ICH), the commonest form in children under 10, and subarachnoid haemorrhage (SAH), which is more common in teenagers.^9^ Arteriovenous malformation (AVM), cavernous angioma, aneurysm and venous sinus thrombosis (VST)^9^ ^10^ are likely vascular pathologies often distinguishable with MR.^10^ Patients with brain tumours, bleeding diatheses, anaemia (including SCD^11^), leukaemia and metabolic disease are also at risk. Compared with ischaemic stroke (fig 1B–F), mortality is higher (8–40%) and recurrence is lower (in a population-based study, 13% for those with medical aetiologies, mainly acutely, and a 5-year cumulative recurrence rate of 13% for those with unoperated AVMs or tumours^9^), although functional outcome may be better, although only a quarter of patients have no physical or cognitive impairment.^12^

Ischaemic stroke is commonly arterial (AIS)^1^ ^13^ ^14^ (figs 1B–D, 3), but cerebral VST is probably under-recognised^15^^–^17 (figs 1E, 4) and should be excluded, particularly in superficial cortical and deep posterior infarcts. The underlying cause or trigger may be a diagnostic clue to the distinction between ATS, VST and stroke mimics^5^ ^18^ (figs 3–5). AIS and VST are associated with death in 6–16%^12^ ^19^ and  3–8% of patients,^15^ ^20^ respectively, higher in those with premorbid conditions (figs 3, 4) and in the critically ill,^19^ while 40–60% of both groups have significant disability.^17^ ^21^ Recurrence rates are age and diagnosis dependent: 3% in neonates,^13^ 6% (3% cerebral) after VST in children 2 years of age with no evidence for recurrence after neonatal VST,^20^ and 10% stroke with an additional 20–35% transient ischaemic events (TIAs) after AIS in childhood.^22^^–^24

## RISK FACTORS

There are similarities and differences between the predisposing conditions and intermediate risk factors for haemorrhagic stroke (figs 1A, 2), AIS (figs 1B–D, 3), VST (figs 1E, 4) and stroke mimics (figs 1F, 5). Certain chromosomal (Down syndrome) and single gene disorders (SCD, homocystinuria) predispose to all (figs 2–5). Approximately half the children presenting with AIS have a predisposing cause^14^ (symptomatic AIS) (fig 3), while the remainder were previously well (cryptogenic AIS); around 80% of both groups have abnormal arterial imaging.^14^ Intermediate risk factors include infections, hypertension, anaemia (including iron deficiency),^14^ ^25^ hyperhomocysteinaemia^26^ and dyslipidaemias including elevated lipoprotein(a).^27^ Thrombophilias such as protein C deficiency, anticardiolipin antibodies and the factor V Leiden and prothrombin 20210 mutations are more frequent in ischaemic stroke populations than in controls^27^ but may be more commonly associated with VST rather than AIS,^14^ ^16^ ^17^ although some appear to be independent risk factors for recurrent cryptogenic AIS.^22^ ^23^

At least a third of cases of childhood stroke occur in the context of infection,^14^ and the importance of prior varicella infection as a risk factor for cryptogenic AIS and haemorrhage has recently been recognised and is apparently secondary to direct infection of the arterial wall, although secondary pathophysiologies, including transient protein C and S deficiency, may play a synergistic role.^14^ ^28^^–^31 High leukocyte count is a risk factor for first haemorrhagic stroke in SCD,^11^ as well as being a risk factor for recurrent AIS.^23^ Host immunity and the cytokine milieu are probably important factors affecting endothelial function and cellular adhesion.

## CHILDHOOD STROKE AND CEREBROVASCULAR DISEASE

The very different nature of the vascular, coagulation and nervous systems in neonates, infants and children means that clinical and radiological presentations are different from adults.

### Cerebrovascular disease associated with haemorrhagic stroke

AVMs (fig 1A4) are defined by the presence of high flow arteriovenous connections without an intervening capillary network (a consequence of abnormal developmental vascular remodeling).^7^ ^9^ Cavernous angiomas probably result from venous hypertension and are multiple lesions (intra- or extracranial) in 13% of sporadic and 50% of familial cases. Aneurysms are relatively rare in children; 10–15% are post-traumatic and a similar proportion are mycotic (fig 1A2, 1A3). VST may also cause intracerebral and subarachnoid haemorrhage. For haemorrhagic strokes, underlying conditions which may require active exclusion include hereditary haemorrhagic telangiectasia, polycystic kidney disease, Ehler-Danlos syndrome type IV, anaemia and hypertension as well as bleeding disorders (fig 2).

### Cerebrovascular disease typically associated with arterial ischaemic stroke (AIS)

#### Extra/intracranial dissection

Arterial dissection (fig 1B) occurs as a consequence of a tear in the intima of an artery leading to extravasation of blood from the lumen into the intermediate layers of the artery and causing local compression, distal embolism or propagation of clot. Clinical signs may therefore falsely localise the pathological arterial territory. Dissection most commonly occurs in the internal carotid and vertebral arteries and risk factors include trauma (apparently minor as well as neck or penetrating pharyngeal injuries, for example falling with a pencil in the mouth) and infection (eg, chronic tonsillitis).^32^^–^34 While most anterior dissections are intracranial (60%), most posterior dissections arise extracranially (60%).^34^

#### Intracranial arteriopathy

Transient cerebral arteriopathy (TCA) (fig 1C3, 1C4) refers to intracranial arterial pathology leading to clinical signs associated with radiological abnormalities that often stabilise and sometimes reverse, although there is a risk of early recurrence.^35^ ^36^ TCA probably represents an inflammatory response to infections such as varicella, Borrelia or tonsillitis.^28^–^31^ ^35^ ^36^ MR typically shows small subcortical infarcts with multifocal arterial wall lesions.^29^ ^30^

Moyamoya is the Japanese for “puff of smoke” and describes a cerebral arteriopathy with bilateral severe stenosis/occlusion of the terminal internal carotid arteries (ICAs) associated with the development of basal collateral vessels.^37^ It may be primary or secondary to SCD, Down syndrome or cranial irradiation. Moyamoya is an independent risk factor for recurrent stroke and TIA,^23^ which are probably reduced after extracranial-intracranial revascularisation.

### Venous sinus thrombosis (VST)

The cerebral veins drain into “superficial” or “deep” cerebral venous sinuses. Sinus blood flow rates are dictated by mean arterial pressure (MAP) and thrombosis therefore occurs more commonly in hypotension. The pathogenesis of VST in neonates remains uncertain but may relate to anatomical distortion during childbirth as well as dehydration and infection. In older children trauma, malignancy or sepsis play a larger role (fig 4). Dehydration, iron deficiency and inherited prothrombotic disorders are additional risk factors.^15^–^17^ ^20^ ^25^ VST may lead to venous hypertension, focal cerebral oedema, haemorrhagic infarction, hydrocephalus and pseudotumour cerebri.^15^^–^17 Although these sinuses may recanalise spontaneously with conservative management (rehydration, antibiotics), acute anticoagulation may be considered,^6^^–^8 as two trials in adults showed reduced mortality and morbidity and a cohort study in children showed reduced recurrence.^20^ Those in whom the risks may be outweighed by the benefits of anticoagulation during periods of risk, for example after relapse of nephrotic syndrome, include children over the age of 2 or with the prothrombin 20210 mutation.^20^

### Other cerebrovascular disease

Vein of Galen malformation (VGAM) is an embryonic arterio-venous fistula occasionally presenting with catastrophic neonatal heart failure which within centres with an experienced multidisciplinary team may be endovascularly palliated^38^; it is usually inappropriate to manage any associated hydrocephalus separately.

Sturge Weber syndrome (SWS) is characterised by a facial capillary haemangioma and venous angiomata of the leptomeninges/choroids associated with intractable epilepsy (which may require hemispherectomy)^39^ and episodic stroke-like episodes leading to progressive hemiplegia and learning disability (reduced with prophylactic aspirin^39^).

## STROKE MIMICS^5^ ^18^

These are diagnoses of exclusion which should only be made after discussion with a paediatric neurologist or intensivist.

### Hemiplegic migraine

There may be a family history. EEG usually shows unilateral slow background activity.

### Acute disseminated encephalomyelitis (ADEM)

The demyelination is usually obvious on MRI. Intravenous methyl prednisolone probably reduces the duration of the illness and perhaps improves long term outcome.

### Reversible posterior leukoencephalopathy syndrome (RPLS)

This is characterised by seizures, disorders of consciousness, visual abnormalities and headaches associated with posterior white matter abnormalities on CT/MRI and has been described after acute chest syndrome in SCD,^40^ after hypertensive encephalopathy and during immunosuppression. The majority of patients make a full clinical and radiological recovery after careful treatment of their underlying condition, although infarction in the parieto-occipital or watershed (fig 1F3) can occur.^40^ If RPLS is associated with hypertension, the blood pressure should be reduced very slowly to avoid precipitous drops and infarction. Vertebrobasilar dissection, which may present with ataxia, visual disturbance or coma rather than hemiparesis (figs 1F1, 3) and which is much commoner in boys,^34^ and VST (figs 1E, 4) are part of the differential diagnosis and should be excluded on emergency imaging as their treatment and prognosis are different.

### Metabolic stroke

There are often clinical clues to the aetiology of metabolic stroke, for example persistent vomiting, hypoglycaemia or diabetes. Organic acidaemias, urea cycle disorders and mitochondrial disorders can cause stroke-like episodes with imaging abnormalities in an atypical vascular distribution. Homocystinuria and Fabry disease are usually associated with cerebrovascular disease.

## CLINICAL PRESENTATION

Although stroke in childhood is relatively rare, its clinical presentation is usually obvious to the paediatrician, who can investigate both obvious and subtle presentations and initiate emergency medical management. Hemiplegia, headache, seizure or altered levels of consciousness may all herald a potentially reversible or lethal medical or surgical stroke emergency. If a stroke syndrome is suspected, initial resuscitation in conjunction with the local anaesthetic/intensive care team should be followed by consultation with regional paediatric neurological services before further imaging, investigation or treatment are planned and instituted. Although stroke is traditionally defined as a neurological deficit lasting for ⩾24 h, many children with a TIA lasting ⩽24 h have had a recent cerebral infarction/haemorrhage on imaging. In addition to the underlying diagnosis (figs 2–5), the time from onset of symptoms to presentation is very useful diagnostically, for example arteriopathy is more likely to present with a stuttering onset,^41^ suggesting the need for imaging to exclude dissection (fig 1), and “thunderclap” headaches may be indicative of a subarachnoid haemorrhage warranting lumbar puncture even if neuroimaging is normal. Stroke mimics may be benign and require no treatment, but in some cases timely intervention prevents neurological disability or death.^5^ ^18^ Emergency MR provides information that can guide management in individual children (figs 2–5). Any financial consequences of over-investigation that this approach may produce will almost certainly be overridden by the potential benefits in terms of reduced healthcare and socio-economic costs resulting from prompt and appropriate treatment.

If immediate transfer is deemed necessary after discussion with the on-call neurosurgeon or paediatric neurologist, liaison with the regional paediatric intensive care unit (PICU) is advisable. Children may present in coma, status epilepticus, or with signs of intracranial hypertension or imminent herniation mandating liaison with local anaesthetic teams/regional PICUs, and appropriate retrieval/transport of children to regional centres for further imaging/management.

## MANAGEMENT AND INVESTIGATION

### Diagnosis independent management

No studies have specifically examined the effect of the loss of cardio-respiratory integrity on stroke outcome in children. However, based on principles which would be applied to the care of any acutely ill child (ABCD, airway, breathing, circulation, disability) including the maintenance of adequate oxygenation (non-invasively estimated by pulse oximetry), cardiac output, systemic and cerebral perfusion pressure, and tight control of blood glucose and body temperature should be the aim. Hypertension should not be treated unless intracranial pressure is monitored if there is a space-occupying lesion, and should only ever be lowered slowly.

Children whose level of consciousness deteriorates should be ventilated and transferred to the nearest neurosurgical/PICU in case they require drainage of a haematoma, ventriculostomy for hydrocephalus or craniectomy for intractable intracranial hypertension. Management may be guided by intracranial pressure monitoring, which should be considered in children who remain sedated and in whom there is radiological/clinical suspicion of a space-occupying lesion. Seizures in the acute phase should be managed aggressively in accordance with conventional algorithms and local guidelines as they significantly increase the cerebral metabolic rate for oxygen and can thus unfavourably affect the substrate supply–demand balance. Consideration should be given to continuous cerebral function monitoring in paralysed children.

### Emergency neuroimaging

Haemorrhagic stroke or AIS with mass effect should be excluded by emergency CT, which might also show some evidence of focal ischaemic damage but often only from 24 h after presentation. If CT is not available immediately at the local site, discussion with a tertiary centre is mandatory and urgent consideration should be given to transfer of the child, even if this can only be achieved safely by intubation and ventilation. The regional PICU should be involved in this decision, as this is an emergency. If immediately available, MR with diffusion weighting has advantages over CT as haemorrhage can be diagnosed or excluded (fig 1A2), RPLS (fig 1F2), hemiplegic migraine (fig 1F4) and ADEM can be distinguished from ischaemic stroke and venous or arterial pathology can usually be identified and this may alter acute management, which in turn may reduce the extent of the eventual infarct. VST may be accompanied by infarction (sometimes haemorrhagic), typically in a parietal, occipital (fig 1E2), frontal or thalamic (fig 1E1) distribution^17^; if the diagnosis is not obvious on plain CT or MR (fig 1E4), emergency CT or MR venography (fig 1E3) should be considered for all strokes unless there is obvious arterial pathology, so that anticoagulation can be considered (see below). For AIS, if MRA is not diagnostic, T1-weighted spin echo of the neck with fat saturation should be performed to exclude dissection (fig 1B3) as, again, these patients should be considered for anticoagulation. Although there is a 1% risk of stroke, highest with intracranial stenosis (fig 1C4), conventional angiography may be required for the diagnosis of small vessel vasculitis, cortical venous thrombosis and sometimes for the diagnosis of dissection (fig 1B4), particularly in the posterior circulation,^34^ as well as for the pre-surgical anatomical definition of moyamoya (fig 1D4), AVM (fig 1A4) or aneurysm.

### Specific measures

#### Haemorrhagic stroke^7^

The management strategy should ensure optimal intravascular volume, normothermia and normoglycaemia. A neurosurgical opinion is mandatory for the discussion of haematoma drainage and the management of complications, including hydrocephalus, vasospasm, perihaematomal oedema and brain shift. Intracranial pressure monitoring and osmotherapy targeted at maintaining an adequate cerebral perfusion pressure may be required. Fluid restriction is not advisable initially but may be initiated if there is evidence of inappropriate ADH release. Vasospasm may complicate subarachnoid haemorrhage, is detectable by transcranial Doppler (TCD) and treatable with calcium channel antagonists. Blood pressure control is a controversial topic as perfusion pressure must be maintained while the risk of recurrent haemorrhage may mandate avoidance of hypertension before definitive vascular treatment. If there is an underlying AVM or aneurysm, the recurrence risk^9^ means that a vascular team with considerable experience should evaluate and decide between the management options (neurosurgery, neuroradiology or stereotactic radiotherapy) once the patient has recovered from the acute phase.

**Box 1 Differential diagnosis in children presenting with acute focal neurological deficit**

All stroke syndromes are potential neurosurgical emergencies and should be discussed with a consultant paediatric neurologist on presentation. Further management and any transfer may involve liaison with the nearest available PICU.

- Acute ischaemic arterial stroke±haemorrhage±mass effect
- Acute venous stroke±haemorrhage±venous infarction±mass effect
- Primary haemorrhagic stroke±mass effect
- Non-accidental injury
-
  - subdural haematoma
  - strangulation with compression of internal carotid artery
- Posterior leukoencephalopathy (hyper/hypotension or immunosuppression)
- Unilateral hemispheric cerebral oedema, for example secondary to diabetes, hyperammonaemia (ornithine carbamoyl transferase deficiency)
- Hemiplegic migraine (but diagnosis of exclusion – migrainous symptoms seen in cerebrovascular disease)
- Post-ictal (Todd’s paresis)
-
  - short duration so neuroimaging essential if persistent
  - children with prolonged seizures may develop permanent hemiparesis with seizures (hemiseizure-hemiplegia-epilepsy)
- Acute disseminated encephalomyelitis
- Brain tumour
- Encephalitis, for example secondary to Herpes simplex (usually have seizures)
- Rasmussen’s encephalitis
- Mitochondrial encephalopathy with stroke-like episodes
- Alternating hemiplegia

#### Ischaemic stroke

Some children presenting with AIS/VST are candidates for acute interventions (figs 3 and 4) after neuroimaging and paediatric neurological consultation.^6^^–^8

##### Transfusion for acute stroke in sickle cell disease

The population with sickle cell disease provides an ideal model for proactive stroke prevention as the majority of strokes are predicted by TCD. Blood transfusion is a mainstay of stroke prevention^42^ as well as acute stroke management. Transfusion should commence within 2–4 h of presentation with neurological deficit; emergency exchange, rather than top-up, transfusion at the time of first stroke appears to be associated with a reduced risk of recurrence.^43^ The aim is to reduce the HbS % to ⩽30% with a haematocrit of >30%. Pathology can include haemorrhage,^11^ VST,^17^ RPLS,^40^ acute necrotising encephalitis and arterial dissection as well as territorial infarction secondary to arterial stenosis and “silent” or covert injury, generally in the watershed regions (fig 1F3) and often associated with transient neurological symptoms and signs rather than overt stroke. If available, emergency MR may guide management.

##### Thrombolysis with tissue plasminogen activator (tPA)

Despite a little published experience,^44^ which is almost certainly biased in favour of positive outcomes, there is no evidence to support the use of tPA in the acute management of childhood stroke.^6^^–^8 Although children may present <3 h after stroke, its rarity, the low sensitivity of CT for acute infarction and the wide differential in this age group means that very few children are diagnosed in the time window described in adult trials of acute intervention in stroke. Generally, thrombolysis is contraindicated. Very occasionally, and only as part of a strict research protocol, thrombolysis, with intravenous tissue plasminogen activator (tPA) within 3 h or intra-arterial tPA within 6 h, may be considered for middle cerebral artery occlusion, or for basilar occlusion, within 12 h, perhaps with balloon angioplasty.

##### Acute anticoagulation or aspirin

The use of anti-coagulation remains controversial. Children are probably at less risk of haemorrhage than adults and there is a case for acute anticoagulation in AIS.^8^ Anticoagulation with low molecular weight heparin followed by warfarin should certainly be considered in children with confirmed VST (for 3–6 months or until complete recanalisation) or extracranial arterial dissection associated with AIS (for 3–6 months or until evidence of vessel healing).^6^^–^8 The use of anticoagulation in patients with cardiac embolism is controversial and management should involve consultants in cardiology and neurology. The use of aspirin probably reduces AIS recurrence^23^ ^45^; aspirin at a dose of 5 mg/kg/day should be considered acutely after AIS, except where there is evidence of haemorrhage, with subsequent long term prophylaxis, particularly if there is persistent vasculopathy, at 3–5 mg/kg/day.^6^^–^8

**Box 2 Emergency imaging for childhood stroke**

Magnetic resonance imaging (MRI) (including diffusion and perfusion), arteriography (MRA), venography (MRV)

- to exclude haemorrhage
- to define extent and territory of infarct
- MRA to define vascular anatomy of circle of Willis and neck vessels
- T1-weighted spin echo of the neck with fat saturation sequence to exclude dissection
- MRV to exclude venous sinus thrombosis
- diffusion imaging to differentiate acute from chronic infarction
- perfusion imaging to demonstrate areas of abnormal cerebral blood flow, blood volume and mean transit time

CT scan to exclude haemorrhage if MRI not available acutely; consider CT venography

Conventional angiography if:

- haemorrhage without coagulopathy and cause not obvious on MRA or MRV
- ischaemic stroke, MRA normal and fat-saturated T1-MRI of the neck does not demonstrate dissection

#### Management of intractable intracranial hypertension

If intracranial hypertension persists or there is evidence of impending herniation despite maximal medical therapy, decompressive craniectomy should be considered for AIS, VST and stroke mimics. Patients with hydrocephalus secondary to large cerebellar infarcts may need ventriculostomy or cerebellectomy.

#### Rehabilitation and follow-up

Physio-, occupational and speech therapists should be available for children soon after stroke as part of the multidisciplinary team. Long term rehabilitation should include cognitive,^46^ as well as physical, domains.

## THE FUTURE

As there is a wide range of differential diagnoses, which may be difficult to recognise,^4^ ^18^ ^47^ and the multidisciplinary expertise available in stroke units has been shown to improve outcome in adults,^48^ it is important that communication between primary, secondary and tertiary services facilitates the child’s pathway through acute diagnosis and treatment as well as longer term rehabilitation and secondary prevention. Further multi-centre, multi-national studies of epidemiology and risk factors for primary and secondary stroke should be urgently undertaken through collaborations such as the International Paediatric Stroke Study. We must then use these observational data to encourage and adequately power randomised interventional studies to establish appropriate evidence based guidelines for the treatment of this potentially salvageable paediatric emergency.
